# Beclin 1 regulates neuronal transforming growth factor-β signaling by mediating recycling of the type I receptor ALK5

**DOI:** 10.1186/s13024-015-0065-0

**Published:** 2015-12-21

**Authors:** Caitlin E. O’Brien, Liana Bonanno, Hui Zhang, Tony Wyss-Coray

**Affiliations:** Cell and Molecular Biology Program, Department of Biology, Stanford University, Stanford, CA 94305 USA; Department of Neurology and Neurological Sciences, Stanford University School of Medicine, Stanford, CA 94305 USA; Center for Tissue Regeneration, Repair, and Restoration, Veteran Administration Palo Alto Health Care System, Palo Alto, CA, 94304 USA; Graduate Program in Neuroscience, Stanford University School of Medicine, Stanford, CA 94305 USA

**Keywords:** Beclin 1, VPS34, Retromer, TGF-β, ALK5, Protein sorting, Receptor recycling, Neurodegeneration

## Abstract

**Background:**

Beclin 1 is a key regulator of multiple trafficking pathways, including autophagy and receptor recycling in yeast and microglia. Decreased beclin 1 levels in the CNS result in neurodegeneration, an effect attributed to impaired autophagy. However, neurons also rely heavily on trophic factors, and signaling through these pathways requires the proper trafficking of trophic factor receptors.

**Results:**

We discovered that beclin 1 regulates signaling through the neuroprotective TGF-β pathway. Beclin 1 is required for recycling of the type I TGF-β receptor ALK5. We show that beclin 1 recruits the retromer to ALK5 and facilitates its localization to Rab11^+^ endosomes. Decreased levels of beclin 1, or its binding partners VPS34 and UVRAG, impair TGF-β signaling.

**Conclusions:**

These findings identify beclin 1 as a positive regulator of a trophic signaling pathway via receptor recycling, and suggest that neuronal death induced by decreased beclin 1 levels may also be due to impaired trophic factor signaling.

**Electronic supplementary material:**

The online version of this article (doi:10.1186/s13024-015-0065-0) contains supplementary material, which is available to authorized users.

## Background

Beclin 1 is a component of the type III phosphatidylinositol-3-kinase (PI3K) complex. In yeast, beclin 1 (Atg6/Vps30) regulates both autophagy [1] and protein sorting [2–4] between the Golgi and vacuole. In this vacuolar protein sorting (vps) pathway, Atg6/Vps30 recruits the retromer complex (composed of Vps35, Vps29, Vps26 and a pair of sorting nexins) to mediate recycling of receptors back to the Golgi. While the role of beclin 1 in autophagy is well established in mammalian cells, we only recently demonstrated a role for mammalian beclin 1 in protein sorting. In microglia, beclin 1 recruits the retromer complex to phagosomes to regulate recycling of the phagocytic receptor CD36 [5], indicating conservation of the sorting function of beclin 1.

Beclin 1 is highly expressed in the nervous system and is essential for neuronal survival. While beclin 1 knockouts are not viable, mice that are heterozygous deficient for beclin 1 experience age-dependent neurodegeneration. Furthermore, beclin 1 deficiency exacerbates amyloid β (Aβ) pathology in a mouse model of Alzheimer’s Disease (AD) [6]. Conditional knockout of beclin 1 in either hippocampal or cerebellar neurons was recently shown to result in rapid neurodegeneration [7]. While basal levels of autophagy are required to prevent neurodegeneration [8, 9], previous studies have not examined a potential role of beclin 1-mediated protein sorting in neuronal survival.

Trophic factors both direct neuronal development and support neuronal survival. Transforming growth factor - β (TGF-β) is a pleiotropic cytokine that mediates diverse effects in the nervous system. During development, TGF-β is both necessary and sufficient for axon specification [10], and is required for synaptic pruning during development of ocular dominance in the lateral geniculate nucleus [11]. In adults, TGF-β signaling is required for the development of immature neurons during neurogenesis [12]. TGF-β also participates in establishment of long-term potentiation (LTP), a correlate of memory formation [13, 14]. In addition to these developmental functions, TGF- β1 is released and exerts neuroprotective effects in response to a variety of injuries, including stroke [15], hypoxia [16], excitotoxicity [16], and Aβ exposure [17, 18]. Inhibition of TGF-β signaling in mice is sufficient to cause age-dependent neurodegeneration [19]. Indeed, the TGF-β pathway is dysregulated in several neurodegenerative diseases including AD [16]. For example, levels of the type II TGF- β receptor are decreased [19], and the downstream Smad proteins are mislocalized in AD [20, 21]. Taken together, these data indicate a critical role for TGF-β signaling in many aspects of nervous system homeostasis.

Signaling through the TGF-β pathway requires interaction of the type I receptor (also known as activin-like kinase 5, or ALK5) and the type II receptor (TBRII) (Additional file 1: Figure S1). Binding of TGF-β by TBRII induces formation of a heterotetrameric complex with ALK5 and results in ALK5 activation [22, 23], which then transduces the signal by phosphorylating Smad2 and Smad3 [24]. In contrast to many other receptor types that are endocytosed only after ligand binding, the TGF-β receptors undergo both constitutive (ligand-independent) and ligand-induced endocytosis [25]. Endocytosed receptors can then either be sorted to the lysosome for degradation and signal attenuation [26] or recycled back to the plasma membrane in a Rab11-dependent manner to maintain signaling competency [25]. Much of our knowledge on TGF-β receptor trafficking and signaling comes from studies on epithelial cells, and receptor trafficking studies in neurons largely focus on the other major receptor types (G-protein coupled receptors, and receptor tyrosine kinases). Given the importance of TGF-β signaling in the nervous system, understanding how TGF-β receptor trafficking is regulated in neurons is critical to our understanding of its developmental and neuroprotective functions. Here we show a novel, autophagy-independent role of beclin 1 in regulating TGF-β signaling in neurons. We demonstrate that beclin 1 recruits the retromer to ALK5 and regulates its recycling, and that loss of beclin 1 results in neuronal death.

## Results

### Beclin 1 is required for neuronal survival in vitro

**Fig. 1:**
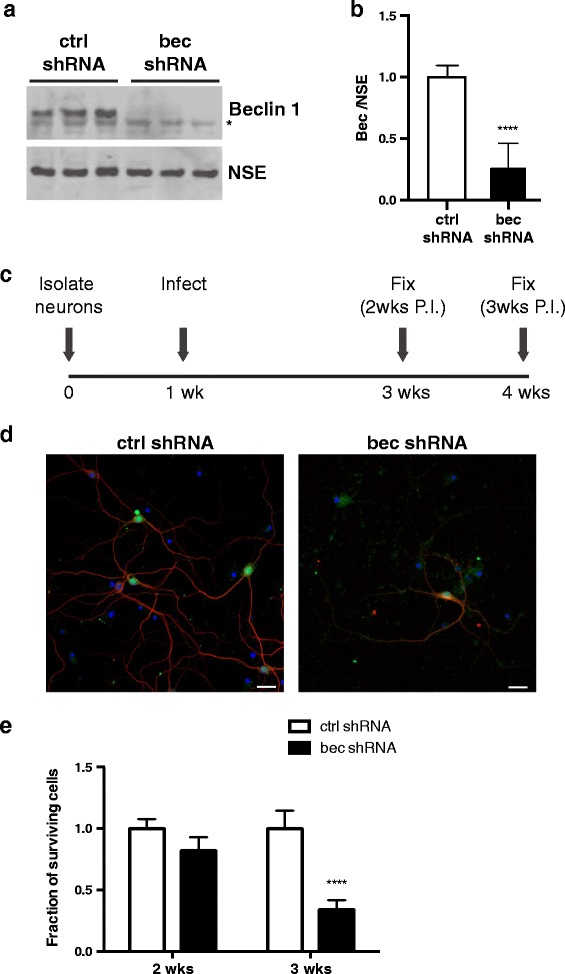
Beclin 1 is required for neuronal survival in vitro. **a** Representative western blot of lysates from primary forebrain neurons isolated from wild type E16.5 embryos. Neurons were infected at 1 week (wk) in culture with a lentivirus encoding a control scrambled or beclin 1-targeted shRNA (ctrl or bec shRNA, respectively) as well as copGFP. **b** Quantification of beclin 1 levels relative to the loading control neuron specific enolase (NSE). Data are combined results of three independent experiments. Data were analyzed by unpaired Student’s *t*-test. **c** Timeline of survival experiment. **d** Representative images of neurons at the 3weeks post-infection (P.I.) time point. Infected cells are GFP positive. Neurons were stained for MAP2 (red) and Hoechst’s dye (blue). **e** Quantification of MAP2^+^ cells at 2 and 3 weeks P.I. The number of surviving MAP2^+^ cells is expressed relative to the number of cells in the ctrl shRNA wells. Data are combined results from 3 independent experiments (*n* = 36 fields/group). Data were analyzed by two-way ANOVA with Sidak’s post-test. ****p ≤ 0.0001., **p ≤ 0.01, ****p ≤ 0.0001. Bar graphs are mean ± SEM.

Given that decreased beclin 1 levels lead to neuronal death in vivo [6, 7], we wanted to establish an in vitro system to study how beclin 1 maintains neuronal survival. To do this, we generated cultures of primary forebrain neurons from wild type CF1 mice and manipulated beclin 1 levels using a lentivirus encoding either a control scrambled (ctrl shRNA) or beclin 1-targeting shRNA (bec shRNA). Infection of primary forebrain neurons resulted in efficient knockdown of beclin 1 by 75 % (Fig. 1a,b). To assess neuronal survival upon beclin 1 knockdown, we followed the experimental design outlined in Fig. 1c. Infected cells were maintained in culture for 2 or 3 weeks post infection (P.I), then fixed and stained for MAP2, a marker of neuronal dendrites. At 2 weeks P.I, there was no significant change in MAP2^+^ neurons (Fig. 1d). However, by 3 weeks post-infection, beclin 1 knockdown resulted in a significant decrease in the number of surviving MAP2^+^ neurons (Fig. 1d, e), supporting previous findings that neurons with decreased levels of beclin 1 degenerate in vivo.

### Beclin 1 localizes to the endosomal system in neurons

In order to determine where beclin 1 may function in neurons, we first looked at localization of beclin 1 in primary neurons. Beclin 1 has been reported to localize primarily to the trans-Golgi network [27] and the endoplasmic reticulum under basal conditions [28, 29] and to nascent autophagosomes during autophagy initiation [30]. However, work from our lab that demonstrates beclin 1 recruits the retromer to phagosomes [5], and others have only recently suggested beclin 1 may function more broadly in the endosomal system [7].

We first confirmed the specificity of the antibody in COS7 cells transfected with FLAG-beclin 1. We stained with antibodies against beclin 1 and FLAG and found a large degree of overlap between the signals (Additional file 1: Figure S2A). We also stained untransfected cells we had infected with our ctrl or bec shRNA. Knockdown of beclin 1 resulted in a significant decrease in staining with the beclin antibody (Additional file 1: Figure S2B, C). Together these results corroborate the specificity of the beclin 1 antibody.

**Fig. 2:**
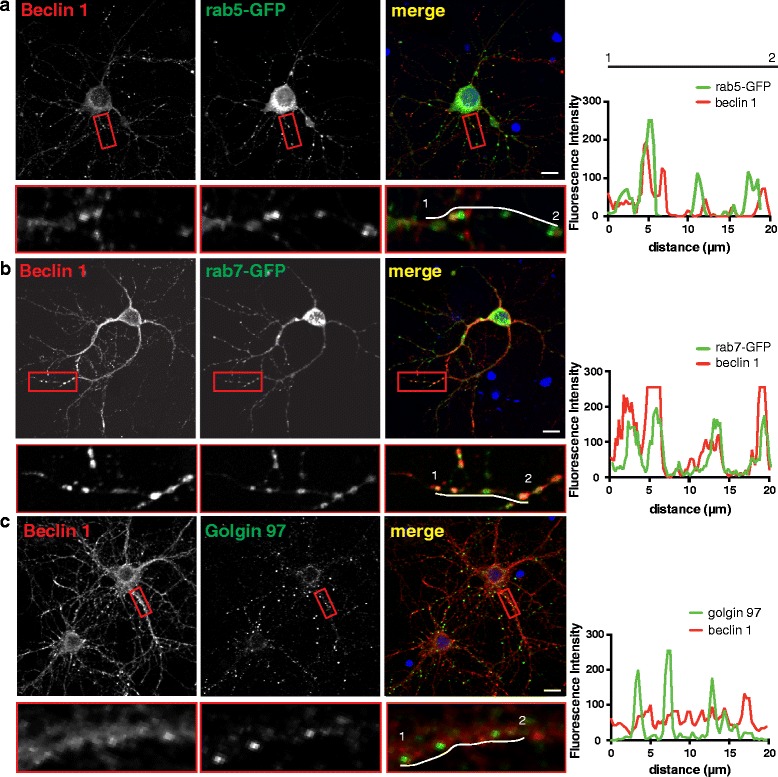
Beclin 1 localizes to endosomes in neurons. Representative images showing colocalization of beclin 1 with (**a**) Rab5-GFP, colocalization coefficient 0.49 ± 0.03, (**b**) Rab7-GFP, colocalization coefficient 0.50 ± 0.03 or (**c**) Golgin 97, colocalization coefficient 0.13 ± 0.01 in primary hippocampal neurons. Regions in the red boxes are enlarged below. Intensity traces (offset white lines) are plotted at right. Scale bars are 10 μm. Colocalization coefficients are the combined results of 2–3 independent experiments (*n* = 28-30 total fields/group).

To determine the localization of beclin 1, we looked for colocalization between beclin 1 and several subcellular markers. In primary hippocampal neurons, beclin 1 was distributed throughout the soma and dendrites where it colocalized with Rab5-GFP, a marker of early endosomes (Fig. 2a, mean colocalization coefficient: 0.49 ± 0.03), and Rab7-GFP, a marker of late endosomes (Fig. 2b, mean colocalization coefficient: 0.50 ± 0.03). Surprisingly, beclin 1 colocalized with these markers to a greater degree than the trans-Golgi marker, Golgin 97 (Fig. 2c, mean colocalization coefficient: 0.13 ± 0.01). This is similar to what we observed in the unpolarized cell line, COS7. In these cells, beclin 1 was found primarily in a perinuclear region (Additional file 1: Figure S3A, B) where it colocalized with Rab5-GFP (Additional file 1: Figure S3C, colocalization coefficient 0.71 ± 0.06), Rab7-GFP (Additional file 1: Figure S3D, colocalization coefficient (0.77 ± 0.08), and to a lesser extent, Golgin 97 (Additional file 1: Figure S3E, colocalization coefficient 0.19 ± 0.02). However, in 20 % of cells, beclin 1 was also found at the cell periphery where it colocalized with Rab5-GFP (Additional file 1: Figure S3B and enlarged region in S3C). These data indicate beclin 1 does indeed localize to endosomes in multiple cell types, making it possible that beclin 1 could play a broader role in regulating receptor trafficking within the endosomal system.

### Beclin 1 regulates levels of the TGF- β receptors

**Fig. 3:**
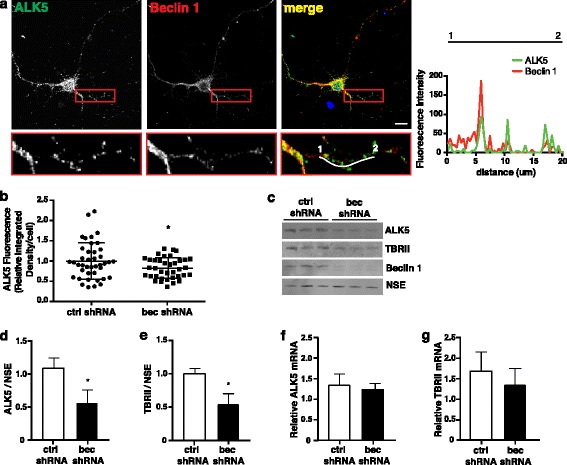
Beclin 1 knockdown reduces ALK5 protein levels. **a** Representative image of CF1 hippocampal neurons stained for beclin 1 and ALK5. Intensity trace (offset white line) is plotted at right. **b** Quantification of ALK5 fluorescence intensity in CF1 hippocampal cells infected with ctrl or beclin 1 shRNA. Only GFP^+^ infected cells were analyzed. Data are combined results from two independent experiments. Each dot represents a single cell (*n* = 37–41 total cells from 20 different fields/group). Data were analyzed by unpaired Student’s *t*-test, ***p ≤ 0.001. **c** Representative western blot of lysates from infected CF1 forebrain neurons. **d** Quantification of ALK5 and **e** TBRII protein levels normalized to NSE. Data are combined results of three independent experiments analyzed by unpaired *t*-test. *p ≤ 0.05. **f** ALK5 and **g** TBRII mRNA levels were measured by RT-PCR and levels were normalized to GAPDH. Relative levels were calculated using the ΔΔCt method. Data are combined results from three independent experiments. Data were analyzed by unpaired Student’s *t*-test. All bar graphs are mean ± SEM.

Given the importance of both beclin 1 and TGF-β signaling to neuronal survival, their disruption in neurodegenerative disease, the ability of beclin 1 to regulate receptor recycling in other contexts, and its localization to endosomes, we hypothesized that beclin 1 may regulate TGF-β receptor sorting and therefore support neuronal survival by regulating signaling through this pathway. We first looked for colocalization between beclin 1 and the type 1 TGF-β receptor ALK5 and found that ALK5 does indeed colocalize with beclin 1 in primary neurons (Fig. 3a, colocalization coefficient 0.59 ± 0.02). We then looked at the effect of beclin 1 knockdown on ALK5. We observed a significant decrease in ALK5 levels upon beclin 1 knockdown by confocal microscopy (Fig. 3b), which we confirmed by western blot (Fig. 3c, d). However, beclin 1 knockdown had no effect on levels of ALK5 mRNA (Fig. 3f). Although we were unable to detect the type II TGF-β receptor TBRII by immunofluorescence, we measured the effect of beclin 1 knockdown on TBRII protein and mRNA levels. Like ALK5, TBRII protein (Fig. 3c, e) but not mRNA (Fig. 3g) levels are decreased by beclin 1 knockdown in primary neurons. These data indicate beclin 1 post-transcriptionally regulates levels of both TGF-β receptors.

### Beclin 1 regulates ALK5 localization with recycling machinery

**Fig. 4:**
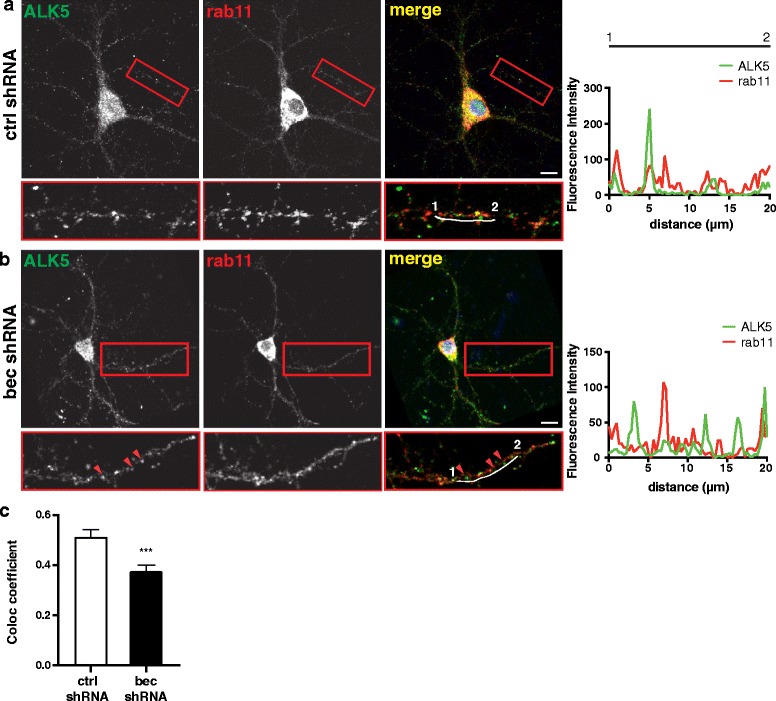
Localization of ALK5 in Rab11^+^ recycling endosomes is beclin 1-dependent. Representative images of neurons infected with (**a**) ctrl shRNA or (**b**) bec shRNA and stained with antibodies against ALK5 (pseudocolored green) and Rab11 (pseudocolored red). Regions in the box were brightened to ease visualization and enlarged below. Intensity traces (white offset lines) from the original image are plotted at right. Red arrowheads indicate ALK5 puncta that don’t colocalize with Rab11. **c** Quantification of the fraction of ALK5 colocalizing with Rab11 in cells infected with ctrl or bec shRNA, expressed as the colocalization coefficient. Data are combined results of three independent experiments (*n* = 27 fields/group). Planned comparison of colocalization coefficients in control and beclin 1 shRNA cells were analyzed by unpaired Student’s *t*-test, ***p ≤ 0.001. Bar graphs are mean ± SEM.

One reason for a decrease in receptor levels could be a failure to recycle these receptors back to the cell surface and their subsequent degradation. To determine if beclin 1 regulates localization of ALK5 in recycling endosomes, we examined the colocalization of ALK5 with a marker of recycling endosomes, Rab11. Rab11 has previously been shown to be required for recycling of the TGF-β receptors [25]. Beclin 1 knockdown decreased the colocalization of ALK5 with Rab11 (Fig. 4).

**Fig. 5:**
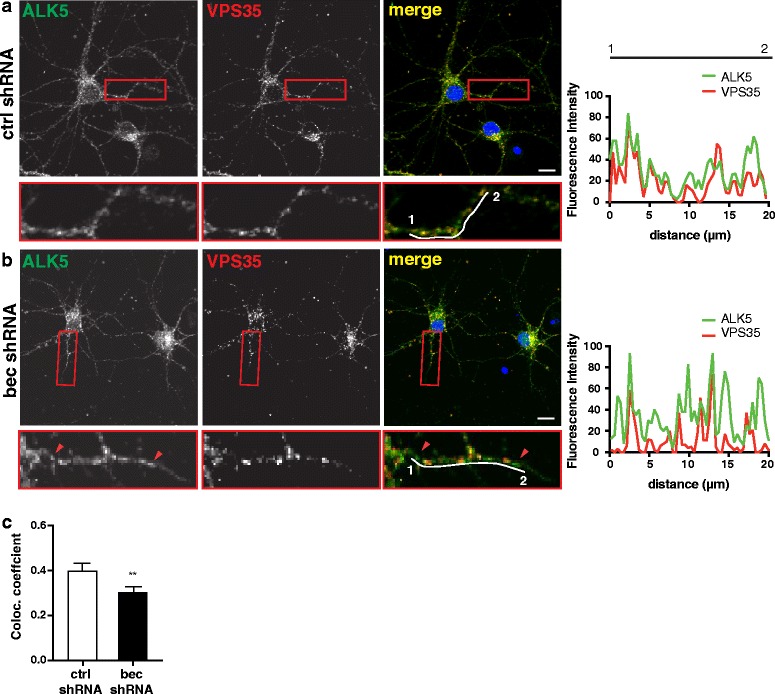
Localization of ALK5 with VPS35 is beclin 1-dependent. Representative images of neurons infected with ctrl shRNA (**a**) or bec shRNA (**b**) stained with antibodies against ALK5 and VPS35. ALK5 is pseudocolored green and VPS35 is pseudocolored red. Regions in the box are enlarged below. Red arrowheads in (**b**) indicate ALK5 puncta that don’t colocalize with VPS35. **c** Quantification of the fraction of ALK5 colocalizing with VPS35 in cells infected with ctrl or bec shRNA, expressed as the colocalization coefficient. Data are combined results of three independent experiments (*n* = 27 fields/group). Planned comparison of colocalization coefficients in control and beclin 1 shRNA cells were analyzed by unpaired *t*-test, **p ≤ 0.01. Bar graphs are mean ± SEM.

In both yeast [31] and microglia [5], beclin 1 mediates receptor recycling by recruiting the retromer, a multi-protein complex composed of VPS35, VPS29, and VPS26, as well as a sorting nexin subcomplex. To determine if beclin 1 recruits the retromer to ALK5, we next analyzed colocalization between ALK5 and the retromer component VPS35. Knockdown of beclin 1 significantly decreased the fraction of ALK5 colocalizing with VPS35 (Fig. 5). These data together suggest that localization of ALK5 with the retromer is in part beclin 1-dependent and that in the absence of beclin 1, sorting of ALK5 into Rab11^+^ recycling endosomes is impaired. Taken together with the effect on ALK5 levels, these results are consistent with the hypothesis that beclin 1 mediates ALK5 recycling and that failure to recycle may lead to receptor degradation.

### Beclin 1 regulates recycling of ALK5

**Fig. 6:**
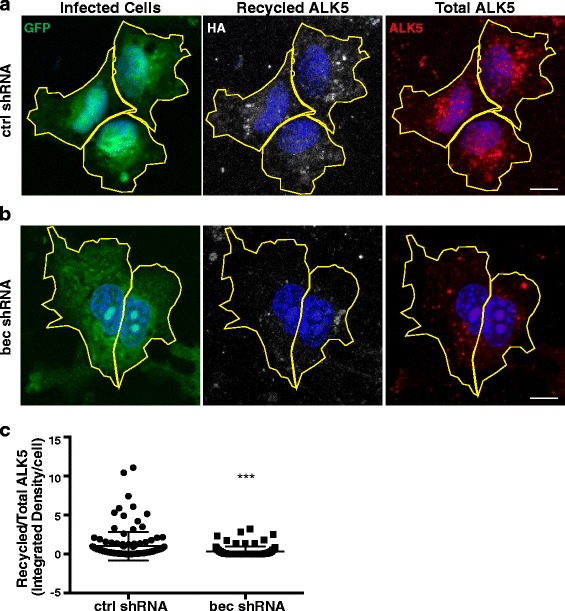
Beclin 1 regulates ALK5 recycling in COS7 cells. Representative images of COS7 cells transiently expressing HA-ALK5 and infected with (**a**) ctrl or (**b**) bec shRNA lentivirus. Only infected (GFP^+^) cells were analyzed. Recycled ALK5 was detected using a mouse anti-HA antibody and a goat-anti-mouse Alexa 647 secondary antibody. Total ALK5 was detected using a rat-anti-ALK5 and goat-anti-rat Alexa 555. **c** Combined results from three independent experiments. Quantification of recycled/total ALK5 from confocal z-stacks. Data is expressed as the ratio of recycled/total ALK5 summed over the z-stack for each cell where each dot represents one cell (*n* = 30 fields/group, n > 85 cells/group). ***p ≤ 0.001. Bar graphs are mean ± SEM

To directly test if beclin 1 regulates ALK5 recycling, we utilized a previously-described antibody-based recycling assay [5, 25]. We expressed a HA-ALK5 tagged plasmid in COS7 cells that had been infected with shRNA lentiviruses and used an anti-HA antibody to detect recycling ALK5. Because TGF-β receptors constitutively recycle and previous studies have shown that these receptors recycle through the same pathway at the same rate in the presence or absence of ligand [25], we performed these experiments in the absence of exogenous TGF-β. We first confirmed that knockdown of beclin 1 does not affect transfection efficiency by transfecting a plasmid encoding mCherry into cells infected with shRNA lentiviruses. Beclin 1 knockdown did not affect transfection efficiency, as quantified by the number of mCherry^+^ GFP^+^ cells (Additional file 1: Figure S4A). Because beclin 1 knockdown decreased total levels of ALK5 (Additional file 1: Figure S4B), cells in the recycling assay were also stained with an anti-ALK5 antibody after fixation, and the results are expressed as the ratio of recycled to total ALK5. Knockdown of beclin 1 resulted in a significant decrease in ALK5 recycling (Fig. 6). These results provide evidence that beclin 1 does indeed regulate ALK5 recycling and support our results showing a decrease in ALK5 localization to Rab11^+^ endosomes.

### Beclin 1 regulates signaling through the TGF-β signaling pathway

**Fig. 7:**
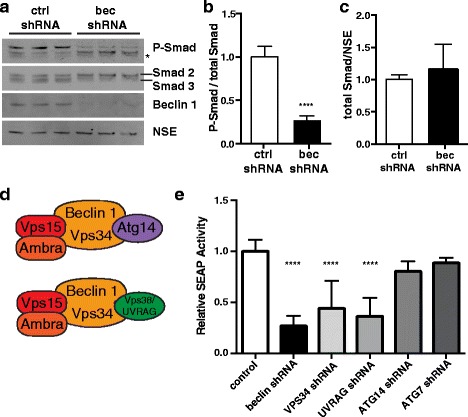
Beclin 1 regulates TGF-β signaling. **a** Representative western blot from infected primary forebrain neurons. Starred band is non-specific. Quantification of (**b**) P-Smad/total Smad levels, and (**c**) Smad relative to NSE from three independent experiments (*n* = 3/group). All bars are mean ± SEM, **p ≤ 0.01, ****p ≤ 0.0001. **d** Mammalian homologs of beclin 1 complexes. Beclin 1 interacts mutually exclusively with Atg14 and UVRAG. In yeast, the ATG14 complex functions specifically in autophagy, while the Vps38 (homologous to UVRAG) complex functions specifically in the vacuolar protein sorting pathway. **e**) SEAP activity in media collected from F11 cells expressing ctrl shRNA or shRNA targeting beclin 1, VPS34, UVRAG, ATG14, or ATG7. Data are combined results from 2–3 independent experiments (*n* = 3/group). Values were normalized to ctrl shRNA levels for each experiment. Data were analyzed by one-way ANOVA with Dunnet’s post-test. All bar graphs are mean ± SEM.

Because beclin 1 reduction affects levels of both TGF- β receptors in neurons, we reasoned that signaling through this pathway would also be affected by beclin 1 knockdown. To test this hypothesis, we measured levels of phosphorylated Smad2 (P-Smad) in lysates from primary mouse neurons. Because these cultures have high basal endogenous levels of TGF-β signaling, we were able to measure Smad phosphorylation in the absence of exogenous TGF-β. Knockdown of beclin 1 resulted in a significant decrease in the ratio of phosphorylated to total Smad2 (to 26 % of control levels, Fig. 7a, b). Total levels of Smad2/3, however, were not changed (Fig. 7c).

To further corroborate these data using a different method, we took advantage of a TGF-β responsive reporter cell line. F11 cells are fibroblasts isolated from TGF-β1^−/−^ mice that have been engineered to stably express secreted alkaline phosphatase (SEAP) under control of the SBE promoter [32]. Treatment of these cells with exogenous TGF-β induces SEAP secretion into the media, which can be collected and assayed for activity. Infection of F11 cells with beclin 1 shRNA lentivirus resulted in a significant decrease in SEAP activity compared to cells infected with the control virus (Fig. 7e). This supports our P-Smad data and suggests that beclin 1 regulation of TGF-β signaling is conserved among cell types.

We also used this method to test the specificity of the effect of beclin 1 knockdown on TGF-β signaling. Beclin 1 is a subunit of the core type III PI3K complex containing the kinase VPS34 and the activating subunits VPS15 and Ambra (Fig. 7d). Beclin 1 also binds ATG14 and UVRAG (a homolog of yeast Vps38) in a mutually exclusive manner. The yeast homologs of these complexes regulate autophagy and protein sorting, respectively [30, 31, 33, 34]. To determine if regulation of TGF-β signaling is specific to beclin 1 and to distinguish between these beclin 1 complexes, we targeted various components of the PI3K complexes and the autophagy pathway. F11 cells were transfected with plasmids encoding shRNA against VPS34, UVRAG, ATG14 or a control shRNA (Fig. 7e). Knockdown of either VPS34 or UVRAG impaired SEAP activity. However, knockdown of ATG14 did not affect SEAP activity. Likewise, knockdown of ATG7, another regulator of autophagy that does not bind beclin 1, had no effect on SEAP activity. Taken together, these results suggest that TGF-β signaling requires a complex of beclin 1, the PI3K VPS34, and UVRAG. These data are consistent with the protein sorting function of this complex in yeast, and indicate that regulation of TGF-β signaling is dependent on beclin 1, but independent of its role in autophagy.

## Discussion

Despite the importance of TGF-β signaling in neuronal development and homeostasis, little is known about the regulation of its receptors and signaling in these cells. We demonstrate here a novel, autophagy-independent role for beclin 1 in regulating TGF-β signaling in both primary mouse neurons and fibroblasts. Our data show that beclin 1 facilitates localization of ALK5 with both the retromer complex and Rab11 in neurons, and provides direct evidence that beclin 1 regulates ALK5 recycling in COS7 cells. The conservation of a beclin 1 function in ALK5 sorting and TGF-β signaling in multiple cell types suggests beclin 1 is fundamental to the regulation of this pathway.

Our results are consistent with a recent report that identified ALK5 as one of over a hundred receptors down-regulated at the surface of cells depleted for either SNX27 or VPS35 [35]. Additionally, an independent report from Yin et al. recently demonstrated a role for the retromer in polarized distribution of TBRII in MDCK cells [36]. TBRII is endocytosed from the apical membrane and recycled through a Rab11-positive recycling endosome to the basolateral membrane in a retromer-dependent manner. While this study did not find a direct interaction between ALK5 and the retromer component VPS26, the observations on the effect of retromer knockdown on ALK5 levels is consistent with our data. These authors found that although ALK5 does not lose its polarized localization upon retromer knockdown (in contrast to TBRII), levels of ALK5 at the basolateral membrane appear to decrease. This is consistent with a role of the retromer in ALK5 recycling, though not polarized localization of this receptor.

The fact that we see only a partial decrease in the colocalization of ALK5 with VPS35 and Rab11 upon beclin 1 knockdown may be due either to incomplete knockdown or to the presence of additional, beclin 1-independent retromer recruitment mechanisms. Indeed, Rojas et al. have previously proposed a model where both Rab5 and Rab7 are required for retromer recruitment to endosomes [37]. Rab5 recruits the PI3K complex to generate PI3P on endosomes, which in turn recruits the sorting nexin subcomplex of the retromer. These authors suggest that the interaction between the sorting nexin and VPS subcomplexes is weak, and an additional interaction of the retromer with Rab7 is required for its localization at the endosome. Our data localizing beclin 1 to both Rab5 and Rab7-positive endosomes make it likely that beclin 1 is indeed part of the PI3K complex that Rab5 recruits. However, additional mechanistic studies are required to test if Rab proteins are sufficient to recruit the retromer to endosomes in the absence of beclin 1.

### A broader role for beclin 1 in protein sorting?

In addition to its function in receptor recycling, beclin 1, as well as UVRAG, has been implicated in downregulation of the EGF receptor (EGFR) via recruitment of the C-VPS/HOPS complex [7, 38]. The recruitment of either the retromer or the C-VPS/HOPS complex by beclin 1 may therefore represent a decision point that determines whether a receptor is recycled or degraded. There are several potential mechanisms by which this may occur. For example, the composition of the beclin 1 complex, or post-translational modifications of its components, may determine which trafficking complex is recruited. Alternatively, modification of the receptor itself (e.g., by ubiquitination), or occupancy of the receptor by its ligand may influence trafficking complex recruitment. Identification of the mechanism by which beclin 1 can recruit multiple protein trafficking complexes and direct receptors into distinct trafficking pathways is an exciting area for future research.

The sorting functions of beclin 1 are all the more intriguing in light of the recent discovery and characterization of beclin 2 [39]. Beclin 2 is present only in mammals, and like beclin 1, functions in both autophagy and protein sorting. Beclin 2 interacts with the GPCR-associated sorting protein, GASP1, to downregulate GPCRs. Future studies are needed to delineate the receptor repertoire for both beclin 1 and beclin 2, as well as the ultimate fates of sorted receptors, and to determine the precise mechanisms by which each member of the beclin family regulates sorting.

### Beclin 1-mediated protein sorting in neurodegeneration

Our lab has previously reported that levels of beclin 1 are decreased in the brains of AD patients, and that heterozygous deficiency of beclin 1 in mice results in neurodegeneration [6]. Since decreased beclin 1 levels have been shown by our lab and many others to impair autophagy, and that autophagy inhibition in neurons is sufficient to cause their degeneration [8, 9], the neuronal death associated with decreased beclin 1 levels was ascribed to autophagy impairment. Recent work from McKnight et al. finds that genetic deletion of beclin 1 in Purkinje cells of the cerebellum or in hippocampal neurons impairs endosomal maturation and results in rapid neurodegeneration [7]. However, this study does not provide a direct link between a role for beclin 1 in the endosomal system and signaling through neurotrophic or neuroprotective pathways.

Our results showing that reduced beclin 1 levels impair signaling in the TGF-β pathway reveal that beclin 1-mediated protein sorting, in addition to its function in autophagy, may be critical for neuronal survival. These data may link the neurodegeneration observed in beclin 1-deficient mice with the age-dependent neurodegeneration observed in mice expressing a kinase-dead TBRII [19]. Furthermore, given that beclin 1 knockdown results in decreased levels of both ALK5 and TBRII, it is intriguing to hypothesize that impaired beclin 1-mediated sorting may cause the decrease in TBRII levels previously observed in AD brain tissue [19]. Future studies should address whether the protein sorting functions of beclin 1 directly contribute to neuronal survival.

TGF-β signaling in AD is particularly interesting for the protection it may provide against some of the negative effects of Aβ exposure. Treatment of primary cultures with TGF-β protects against Aβ-induced neurotoxicity [17, 18], while pharmacological inhibition of ALK5 in rats exacerbates neurotoxicity of Aβ oligomers injected into the hippocampus [17]. Inhibition of beclin 1-mediated TGF-β signaling in AD may therefore sensitize neurons to Aβ exposure and contribute to overt neuronal death. Prior to neuronal demise, TGF-β may also protect against cognitive changes induced by Aβ. Aβ exposure impairs LTP in hippocampal slices [40], which may lead to the deficits in learning and memory observed in mouse models of AD [41]. TGF-β, however, can positively modulate LTP. Treatment of hippocampal slices with TGF-β prior to a weak stimulus converts early LTP to late-LTP, while pharmacological inhibition of ALK5 impairs LTP and cognitive performance in mice [14]. A decrease in beclin 1 levels and subsequent inhibition of TGF-β signaling early in disease may therefore contribute to impaired LTP and decreases in cognitive function prior to neuronal death. Future studies should address whether decreased levels of beclin 1 impair LTP in both wild type and AD mouse models, as well as the potential therapeutic benefit of increased TGF-β signaling in protecting against Aβ-induced cognitive impairments. Given that beclin 1 reduces levels of the TGF-β receptors, therapies that target TGF-β signaling downstream of the receptors, rather than simply increasing ligand levels, may prove more efficacious.

Our work presented here fits with an emerging literature that links dysregulation of protein trafficking pathways with neurodegeneration. Levels of VPS35 are decreased in AD [42], and mutations in VPS35 have been identified in patients with Parkinson’s Disease (PD) [43, 44]. Mutations in the VPS10 receptor family proteins SorLA and SorCs, well-known retromer cargo that mediate trafficking of the amyloid precursor protein, have also been linked to AD [45–47]. Additionally, variants of CHMP2B, a component of the ESCRT machinery that functions in multivesicular body formation, are associated with frontotemporal dementia [48]. Looking more broadly within the endosomal system, mutations in Rab7 are associated with Charcot-Marie-Tooth Disease [49]. In addition to alterations in the trafficking machinery itself, variants in the microglial phagocytic receptor Trem2 that have been associated with neurodegenerative diseases including AD, PD, amyotrophic lateral sclerosis, and frontotemporal dementia impair maturation of this receptor [50]. These studies together strongly suggest that the proper endosomal trafficking of receptors in multiple cell types in the brain is critical for the maintenance of this organ and the prevention of neurological disease.

## Conclusions

Neurodegenerative diseases such as Alzheimer’s Disease are associated with alterations in multiple pathways important for neuronal homeostasis, including autophagy, growth factor signaling, and protein sorting. Because beclin 1 functions in both autophagy and protein sorting, it represents an intriguing therapeutic target for neurodegenerative disease. Indeed, lentiviral delivery of beclin 1 in a mouse model of Parkinson’s Disease was shown to protect against the loss of neuronal markers [51], and in an AD model reduced amyloid pathology [6]. While these effects were attributed to increased autophagy, it is possible that enhanced protein sorting also contributed to this rescue. Our work presented here links beclin 1 with the TGF-β pathway. A decrease in beclin 1 levels, as previously observed in AD [6], leads to impaired ALK5 recycling and deprives neurons of neuroprotective TGF-β signaling. Future studies should determine the full extent of beclin 1-mediated protein sorting, and its contribution to neuronal survival. In particular, identification of the full receptor repertoire beclin 1 regulates may reveal additional trophic factor or other signaling pathways and provide a deeper insight into the consequences of altered beclin 1 levels. Knowledge of both the autophagy and protein sorting functions of beclin 1 should inform strategies to target beclin 1 in neurodegenerative disease.

## Methods

### Cell culture and primary neuron isolation

Single cell-suspensions of primary hippocampal and cortical neurons were isolated at E16.5 from CF1 pregnant mothers (Charles River) following the protocol described in Fath et al. [52]. Cells were seeded onto 24-well plates with or without coverslips coated with 0.1 mg/mL poly-L-lysine in boric acid pH 8. Neurons were maintained in neurobasal medium + B27. Hippocampal neurons were used for microscopy (50,000 cells/well) only due to their limited numbers, while cortical neurons were used for western blotting (200,000 cells/well). COS7 and mouse fibroblast (MFB) F11 cells were cultured in DMEM media supplemented with 10 % fetal bovine serum. For TGF-β treatments in F11 cells, cells were washed 2× in PBS and incubated with 1 ng/mL TGF-β1 (R&D Systems) or TGF- β3 (National Institute for Biological Standards and Control #98/608) in serum-free media for 1 h.

### Plasmids and lentiviruses

The lentivirus knockdown plasmids contain shRNA targeting mouse beclin 1 at nucleotides 405–423 [5] or a scrambled control shRNA in the pSIH-H1 vector from SBI System Biosciences. This plasmid also contains a copepod GFP (copGFP) to monitor infection efficiency. All lentiviruses were generated by the Stanford Neuroscience Gene Vector and Virus Core. Plasmids for VPS34, UVRAG, ATG7, and Atg14 shRNA were obtained from Santa Cruz Biotechnology Inc. Plasmids were provided as pools of three 19–25 bp target shRNA. The Rab5-GFP and Rab7-GFP plasmids were generously provided by Dr. Craig Garner.

### Cell transfection and viral transduction

Primary cells were infected at 7 DIV at an MOI of 20 in neurobasal medium + B27. Virus was removed 16–20 h later and cells were analyzed 7 days post infection.

F11 or COS7 cells were plated in media containing 8 μg/mL polybrene for 1 h prior to virus addition at an MOI of 50. Virus was removed after 16–20 h and cells were analyzed 48 h post infection. For transfection, cells were transfected using lipofectamine 2000 (Thermo Fisher).

### Antibodies for western blotting (WB) and immunocytochemistry (ICC)

Beclin 1 (WB 1:500, BD Biosciences #612113), Beclin 1 (ICC 1:500, Anaspec #54229), Phospho-Smad2 (WB 1:500, Millipore # AB3849), Smad 2/3 (WB 1:500, Cell Signaling Technology # 3102S), TGF beta receptor 1 (ALK5) (WB 1:500, Abcam # ab31013), TGF beta receptor 1 (ALK5) (ICC 1:100, R&D Systems # MAB5871), TGF beta receptor 2 (TBRII) (WB: 1:500, Santa Cruz Biotechnologies # sc-220), VPS35 (ICC 1:500, Abcam # ab10099), Rab11 (ICC: 1:500, Cell Signaling Technology #3539S), Neuron Specific Enolase (WB 1:1000, Thermo Scientific Pierce #MA1-16696), Golgin 97 (ICC 1:250, Thermo Fisher #A-21270), MAP2 (ICC 1:1000, Abcam #ab32454), HA.11 Clone 16B12 (Recycling 1:100, Fisher #NC9693348).

### Immunocytochemistry and confocal microscopy

CF1 primary hippocampal cells were grown on coverslips coated with 100ug/ml poly-L-lysine in 100 mM boric acid (pH8). Cells were then fixed in 4 % paraformaldehyde and permeabilized with 0.02 % triton X-100. Coverslips were blocked for 1 h in 5 % milk in TBST, and then incubated with primary antibodies overnight at 4 °C in a humidified chamber. After washing, coverslips were incubated with species-specific Alexa-dye conjugated secondary antibodies for 1 h at RT. Cell nuclei were stained with Hoechst’s dye (Thermo Fisher). All microscopy was performed on a Zeiss LSM 700. Images were collected using the Zeiss Zen software. Mander’s colocalization coefficients were generated using the Zen software. Fluorescence integrated density measurements were made in ImageJ software (version 1.48, NIH).

### Western blotting

Cells were lysed in RIPA buffer containing Halt Protease and Phosphatase Inhibitor Cocktail (Roche) and total protein concentrations were determined with a BCA Protein Assay Kit (Thermo Scientific). 10 μg total protein/sample was loaded into precast 4–12 % bis-tris gels and run with MES buffer (Invitrogen). Gels were transferred onto 0.2 μm nitrocellulose (BioRad) and incubated with antigen-specific primary antibodies at 4 °C overnight. For LiCor detection, membranes were incubated with species-specific IR Dye antibodies (LiCor) and scanned on an Odyssey Infrared Imager. Bands were quantified using ImageJ software (version 1.48, NIH).

### Quantitative PCR

RNA was isolated from CF1 cortical cells using the Qiagen RNeasy Mini Kit and treated with DNase I (Thermo Fisher) to degrade genomic DNA. cDNA was generated from 1 μg RNA using the SensiFast cDNA Synthesis Kit. Equal amounts of cDNA were used for RT-PCR. The following primers were used:

- ALK5: F – CAGCTCCTCATCGTGTTGGT
  - R - GCACATACAAATGGCCTGTCTC
- TBRII: F -CCTCACGAGGCATGTCATCAG
  - R - ACAGGTCAAGTCGTTCTTCACTA
- GAPDH: F – AGGTCGGTGTGAACGGATTTG
  - R - TGTAGACCATGTAGTTGAGGTCA.

RT-PCR was carried out on a Roche LightCycler 480 Instrument II using the SYBR Green I Master Mix (Roche) to detect DNA levels. The ΔΔCt method was used to quantify relative mRNA amounts in each sample.

### Neuron survival assay

CF1 hippocampal neurons (7 DIV) cultured on coverslips were infected with control or beclin shRNA lentivirus and cultured either for an additional 2 or 3 weeks. Cells were fixed as above and stained for MAP2. Coverslips were imaged using a 10× objective by confocal microscopy. Three randomly selected fields were imaged per coverslip (3 coverslips/condition) and the number of MAP2-positive neurons were counted (9 fields/condition). Results are expressed as relative number of cells/field.

### SEAP assay

MFB-F11 cells stably expressing the SBE-SEAP reporter construct [32] were plated at a density of 40,000 cells/well in 96-well plates and allowed to adhere overnight. Cells were washed twice with PBS and treated with or without 1 ng/mL TGF-β1 (R&D Systems) overnight. Media was collected and assayed for SEAP activity using the Roche chemiluminescent SEAP Reporter Gene Assay.

### Receptor recycling assay

Receptor recycling assay was performed as previously described [25]. COS7 cells were plated onto poly-L-lysine coated coverslips and infected with lentivirus as described above. Forty-eight hours after infection, cells were transfected with a plasmid encoding HA-tagged ALK5. Twenty-four hours after transfection, cells were incubated in 1 % goat serum (the source of the secondary antibody) in DMEM for 15 min on ice to block. To measure recycling, cells were incubated with anti-HA antibody (1:100 in 1 % goat serum + DMEM) for 1 h at 37C. Cells were then acid washed on ice with cold DMEM at pH 2.0, then with cold PBS. Cells were then incubated with secondary antibody for 1 h at 37C. Cells were again acid washed with cold DMEM at pH 2.0 and washed with cold PBS. Cells were then fixed with 4 % PFA, permeabilized with 0.2 % triton in PBS, and washed. After permeabilization, cells were blocked for 1 h in 5 % milk, and then stained for total ALK5 (1:250 in 5 % milk) as described above. Image z-stacks were collected by confocal microscopy. Infected (GFP+) cells were outlined and measurements were made for all GFP+ cells in an image using ImageJ software (version 1.48, NIH). Fluorescent signals of anti-HA (recycled) and anti-ALK5 (total) were thresholded and the fluorescent integrated density was measured for each image in the stack for every cell. The ratio of surface or recycled to total ALK5 for each cell was then taken, and summed for all images in the stack. Only cells with ALK5 intensity density over 10/cell area and HA intensity density over 1/cell area were analyzed.

### Statistics

All statistical analyses were conducted using the Prism6 (GraphPad Software). Differences between control and treatment conditions were calculated using a Student’s unpaired *t* test, one-way ANOVA with Dunnet’s post-test to compare test conditions to control, or a two-way ANOVA with Sidak’s post-test for groups with multiple variables. All bar graphs are presented as mean ± SEM and *p* values less than 0.05 were considered significant. Statistical details for each experiment are indicated in the figure legends.

## Additional file

Additional file 1:
**Figure S1**: The TGF-β signaling pathway. **Figure S2**: Specificity of the beclin 1 antibody. **Figure S3**: Beclin 1 localization in COS7 cells. **Figure S4**: Control data related to the recycling assay (Fig 6). (PDF 9708 kb)

## Abbreviations

Aβ: amyloid beta
AD: alzheimer’s disease
ALK5: activin-like kinase 5
ATG: autophagy related gene
bec: beclin 1
C-VPS/HOPS: class C vacuolar protein sorting/homotypic fusion and protein sorting
CD36: cluster of differentiation 36
CHMP2B: charged multivesicular body protein 2b
CNS: central nervous system
ctrl: control
EGFR: epidermal growth factor receptor
ESCRT: endosomal sorting complexes required for transport
GASP1: GPCR associated sorting protein 1
GFP: green fluorescent protein
GPCR: G-protein coupled receptor
LTP: long term potentiation
MAP2: microtubule associated protein 2
MDCK: madin-darby canine kidney
NSE: neuron specific enolase
P.I: post infection
PI3K: phosphatidylinositol-3-kinase
PD: parkinson’s disease
P-Smad: phosphorylated smad
SBE: smad binding element
SEAP: secreted alkaline phosphatase
SNX: sorting nexin
SORCS: sortilin-related VPS10 domain containing receptor
SorLA: sorting protein-related receptor
TBRII: type II TGF-β receptor
TGF-β: transformation growth factor beta
UVRAG: UV radiation resistance associated gene
VPS: vacuolar protein sorting
