# Supplementary material for: Beclin 1 regulates neuronal transforming growth factor-β signaling by mediating recycling of the type I receptor ALK5
Source: Mol Neurodegener. 2015 Dec 21;10:69. doi: 10.1186/s13024-015-0065-0 (PMC4687091; doi:10.1186/s13024-015-0065-0)
Supplement: Additional file 1: — Figure S1: The TGF-β signaling pathway. Figure S2: Specificity of the beclin 1 antibody. Figure S3: Beclin 1 localization in COS7 cells. Figure S4: Control data related to the recycling assay (Fig 6). (PDF 9708 kb) [file 13024_2015_65_MOESM1_ESM.pdf]

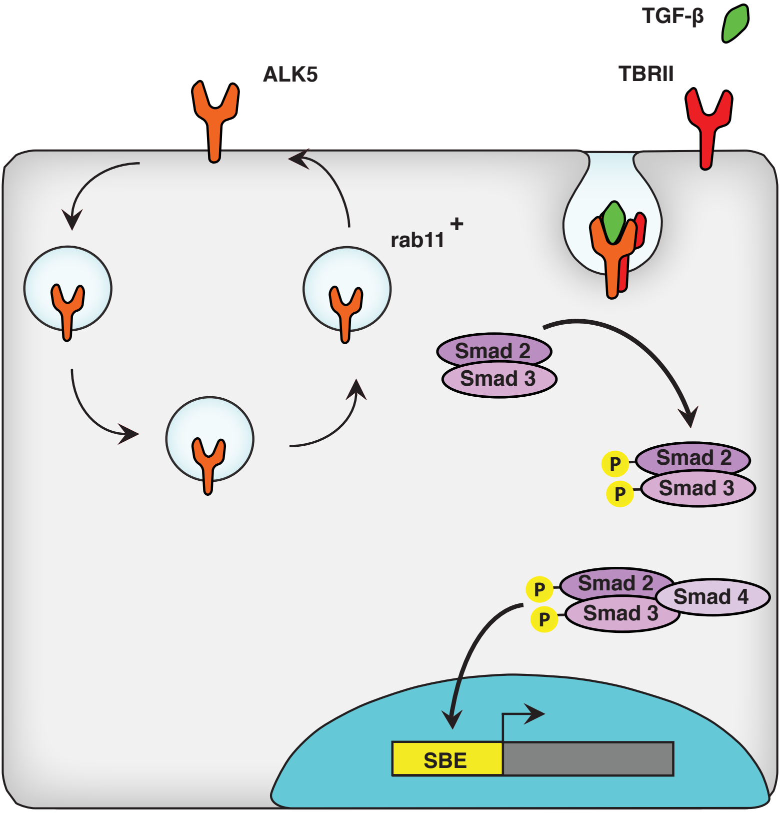

Figure S1: The TGF-β signaling pathway.

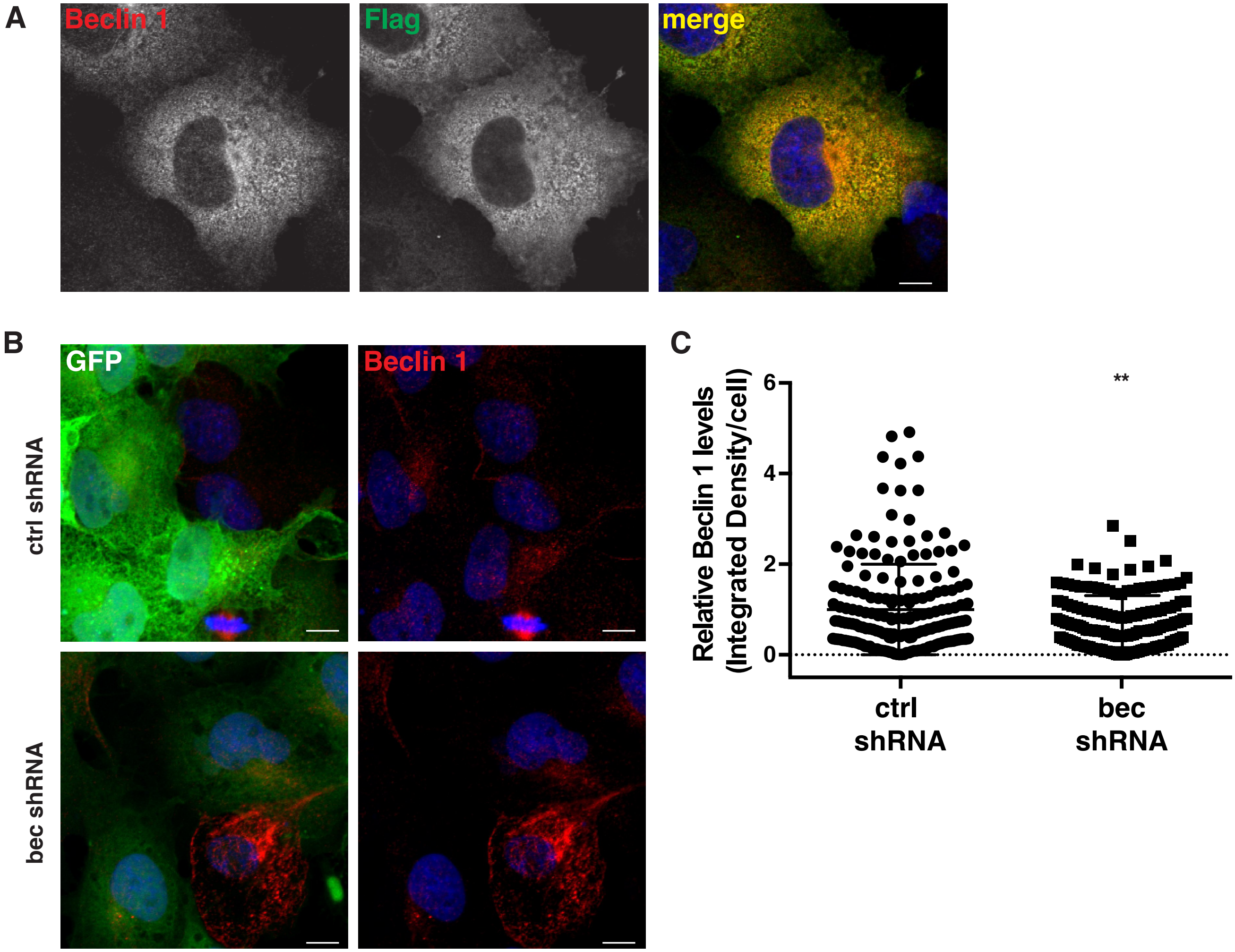

Figure S2: Specificity of beclin 1 antibody

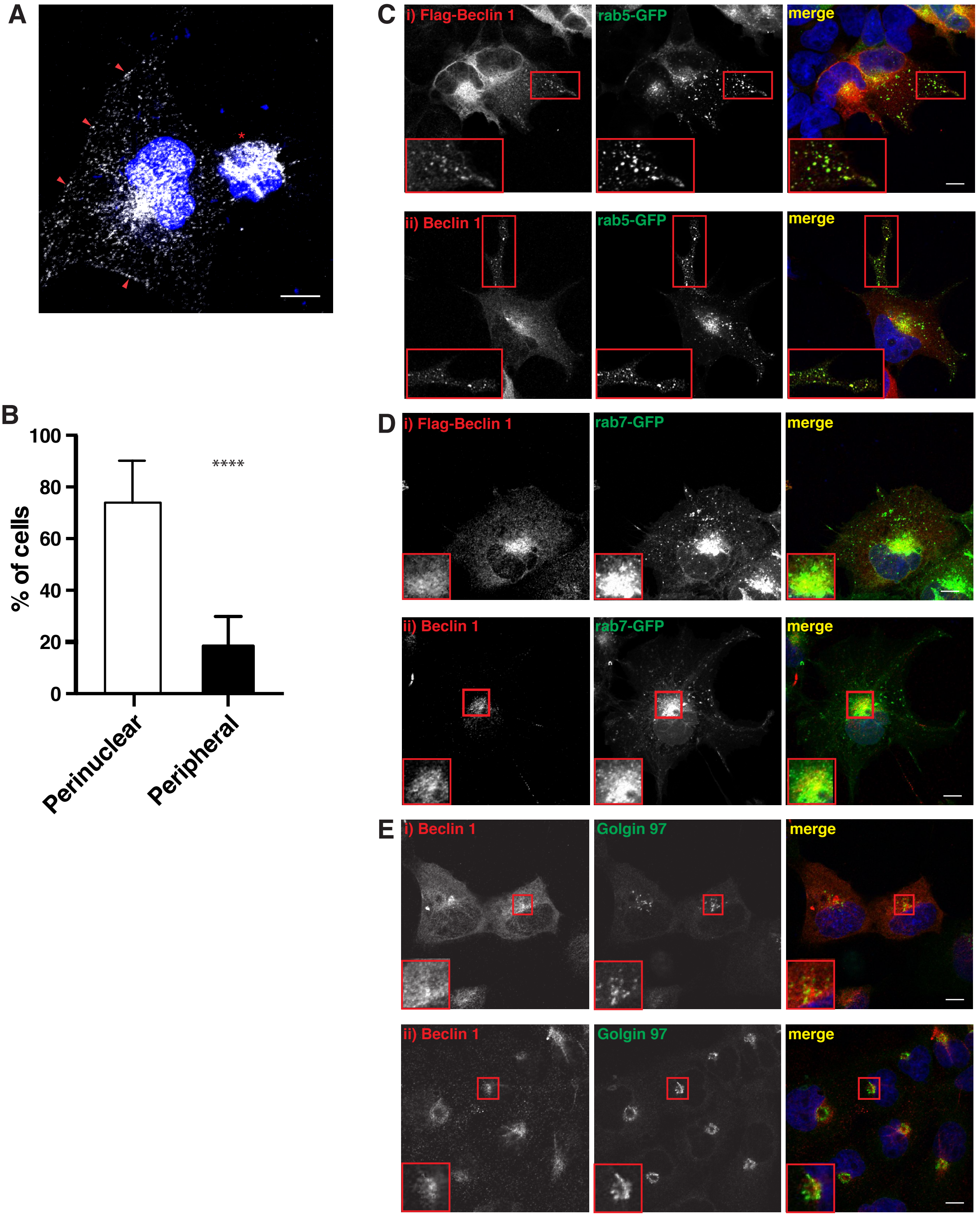

Figure S3: Beclin 1 localization in COS7 cells.

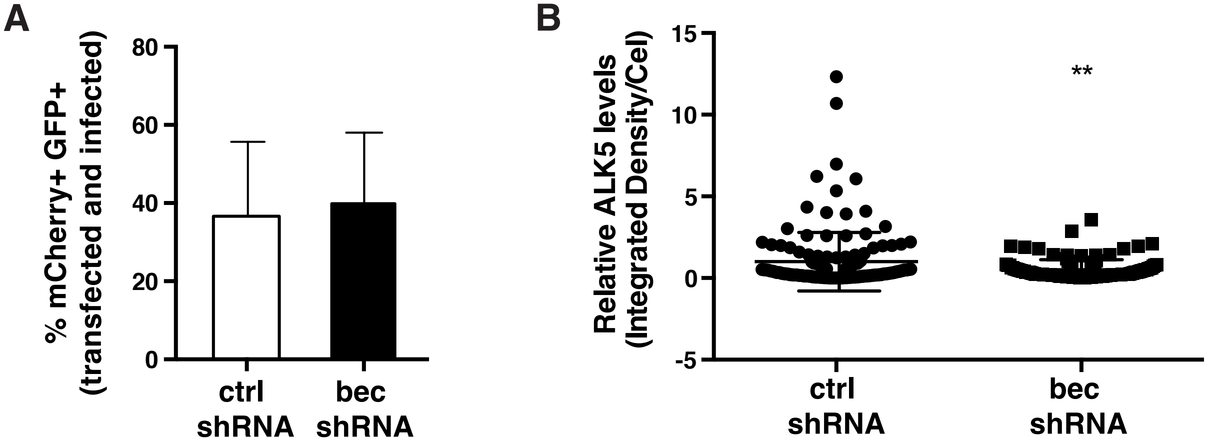

Figure S4: Control data related to the recycling assay (Figure 6).

Figure S1: The TGF-β signaling pathway. TGF-β binds to the type II receptor (TBRII), which then forms a heterotetrameric complex with the type I receptor, ALK5. TBRII is a constitutively active serine/threonine kinase that phosphorylates ALK5, thereby activating it. ALK5 is also a serine/threonine kinase that phosphorylates Smad2 and Smad3. Smad2&3 form a complex with Smad4, and translocate to the nucleus to induce transcription of genes under control of the Smad-binding element (SBE). Both ALK5 and TBRII are constitutively endocytosed in the absence of ligand and can recycle back to the cell surface in a Rab11-dependent manner.

Figure S2: Specificity of beclin 1 antibody. A) COS7 cells were transfected with Flag-tagged beclin 1 and stained for Flag and beclin 1. Representative image showing colocalization of beclin 1 antibody with flag antibody. B) Representative images of COS7 cells infected with lentivirus expressing control scrambled or beclin 1 shRNA and stained for beclin 1. GFP- green; beclin - red; Hoescht's dye – blue. Note the difference in beclin intensity in the infected GFP^+ cell compared to the uninfected cell in the same field. C) Quantification of beclin 1 staining in infected cells. GFP images were thresholded to create a mask for infected cells and integrated density (fluorescence area x fluorescence mean) of beclin 1 staining was measured in infected cells. Data are combined results from two independent experiments. Each dot represents one cell ($n > 100$ cells/group).

Figure S3: Beclin 1 localization in COS7 cells. A) Representative image showing distribution of beclin 1 in COS7 cells. Star indicates perinuclear beclin 1 localization, while arrowheads indicate beclin 1 at the cell periphery. Scale bar is 25μm. B) Quantification showing the percentage of cells with either perinuclear or peripheral beclin 1 localization. Results are combined from 2 independent experiments. Approximately 600 cells were scored in total. C, D &E) Representative images showing colocalization of Rab5-GFP (C), Rab7-GFP (D), or Golgin 97 (E) with Flag-beclin 1 (i) or endogenous beclin 1 (ii). Regions in the red boxes are enlarged in inset. In C), the box highlights peripheral regions where beclin 1 colocalizes with Rab5-GFP. In D) & E) the boxes highlight perinuclear regions where beclin 1 colocalizes with Rab7-GFP or Golgin 97, respectively. Colocalization coefficients (reported in text) were quantified from images of endogenous beclin 1 ($n \geq 10$ fields/group). Scale bars are 10μm.

Figure S4: Control data related to the recycling assay (Figure 6). A) To ensure that a decrease in surface or recycled HA-ALK5 is due to impaired receptor trafficking and not an effect of beclin 1 KD on transfection efficiency, additional COS7 cells were transfected alongside cells for the recycling experiments with a plasmid encoding mCherry. Cells were fixed at the same time as the cells used for the recycling experiments. The number of $mCherry^+$ cells were then counted and expressed as the % of GFP^+ infected cells. Data are combined results from two independent experiments ($n > 200$ cells/group). B) Total ALK5 levels (integrated density)/cell in GFP^+ cells from recycling experiments. Each dot represents one cell. Data are combined results from three independent experiments. ** $p \leq 0.01$. Bar graphs are mean ± SEM.
